# Adherence to Dietary Guidelines among Women with and without Gestational Diabetes: Evidence from the Growing up in New Zealand Study

**DOI:** 10.3390/nu14102145

**Published:** 2022-05-21

**Authors:** Robyn L. Lawrence, Clare R. Wall, Frank H. Bloomfield

**Affiliations:** 1 The Liggins Institute, The University of Auckland, Auckland 1023, New Zealand; robyn.lawrence@auckland.ac.nz; 2 Disciple of Nutrition and Dietetics, Faculty of Medical and Health Sciences, The University of Auckland, Auckland 1023, New Zealand; c.wall@auckland.ac.nz

**Keywords:** gestational diabetes, dietary guidelines, diet, pregnancy

## Abstract

Diet is thought to play a role in the development and management of gestational diabetes mellitus (GDM). Dietary guidelines provide practical recommendations for achieving nutrient requirements and mitigating the risk of chronic disease. The aim of this study was to describe the adherence to dietary guidelines by women with and without GDM and determine whether adherence is associated with the development of GDM. Adherence to Ministry of Health food group recommendations was assessed in 5391 pregnant women participating in the Growing Up in New Zealand study. A food frequency questionnaire (FFQ) administered during pregnancy provided dietary data. The presence of GDM was determined using diagnostic coding in clinical data and blood glucose results. A quarter of women did not meet any food group recommendations. There were no significant differences in the number of food group targets met by women with or those without GDM. Meeting food group recommendations was not associated with odds of having GDM in adjusted analyses. This study found adherence to dietary recommendations is poor in both women with and without GDM and no association between adherence to food group recommendations and the development of GDM. Greater support is required to assist women to achieve food and nutrition recommendations.

## 1. Introduction

Pregnant women have higher nutritional demands in order to meet their needs and those of their growing foetus [1,2]. Diet prior to and during pregnancy can have significant health implications for both mother and baby [3]. Gestational diabetes mellitus (GDM), carbohydrate intolerance first diagnosed during pregnancy, is one of the most common complications of pregnancy [4]. GDM is estimated to effect 6% of pregnancies in New Zealand [5]. Risk factors for GDM include increased maternal age, family history of diabetes, ethnicity, higher maternal pre-pregnancy body mass index (BMI) and diet [6,7,8]. Dietary patterns characterised by high intakes of vegetables, fruit, legumes, fish, wholegrains, nuts, seeds and vegetables have been associated with a reduced risk of developing GDM [9,10,11,12,13] whilst dietary patterns characterised by high intakes of red and processed meats, sugar-sweetened beverages, French fries, pizza, high-fat dairy, refined grains, cakes, biscuits and confectionery have been associated with an increased risk of GDM [11,12,13]. Although dietary patterns give an indication of the diet as a whole, dietary recommendations are commonly based on individual food groups. Food and nutrition guidelines provide practical recommendations for specific populations to assist them in achieving estimated nutrient requirements, thereby reducing the risk of developing chronic disease [14]. Greater adherence to food and nutrition guidelines in China has been reported to be associated with a reduced risk of developing GDM [15]. The Growing Up in New Zealand (GUiNZ) study, the largest study of dietary intake of pregnant women in New Zealand, found only 3% of women met the recommended number of daily servings for each of the four food groups and 24% of women did not meet any of the recommendations [16]. How the diet of women with GDM compares to food and nutrition recommendations and whether greater adherence to recommendations is associated with a reduced risk of developing GDM in New Zealand is unknown. The aim of this study was to describe the proportion of women with GDM meeting the Ministry of Health’s Food and Nutrition Guidelines for Pregnant and Breastfeeding Women (FNGPB), determine whether there are differences in adherence between women with and without GDM and whether a diet that adheres to these guidelines is associated with a reduced odds of having GDM.

## 2. Materials and Methods

Pregnant women with an estimated due date between 25 April 2009 and 25 March 2010, residing in a demarcated area in the North Island of New Zealand and enrolled in GUiNZ (www.growingup.co.nz), an ethnically diverse, longitudinal pre-birth cohort, were eligible to participate. The area specified for recruitment was selected for its ethnic, sociodemographic and environmental diversity to recruit a study population broadly generalizable to the rest of New Zealand [17]. Ethical approval was granted on 1 August 2008 by the Ministry of Health Northern Y Regional Ethics Committee (NTY/08/06/055); participating women provided informed consent. Methodology and reporting is consistent with STROBE guidelines [18].

Data on maternal demographics, health and pregnancy history, smoking status, diet and physical activity were collected in-person interviews by trained interviewers. The antenatal interview was completed by 6822 women and 6657 consented to the use of their national health identifier to access their health records. Women interviewed after the child’s birth were excluded from this study in order to minimise the effect of recall bias on diet during pregnancy. The mean gestational age at the time of the antenatal data collection interview was 31 (standard deviation (SD) 4) weeks (*n* = 5584 for whom data on expected due date were available). Most women (*n* = 4365, 78%) completed the interview during the third trimester. Coding criteria used by Statistics New Zealand [19] were used to categorise self-reported ethnicity into one of six categories: European; Māori; Pacific Peoples; Asian; Middle Eastern/Latin American/African (MELAA), and Other. The ‘MELAA’ and ‘Other’ ethnic groups were merged into ‘Other’ due to small numbers. The New Zealand Deprivation Index (NZDep06) [20], was used as a measure of social deprivation and is made up of deciles from 1 (least deprived) to 10 (most deprived). Pre-pregnancy BMI was calculated using self-reported pre-pregnancy weight and height. The International Physical Activity Questionnaire (IPAQ) [21] was used to measure physical activity levels. A semi-quantitative 44-item food frequency questionnaire (FFQ) assessing intake over the past four weeks was administered during the antenatal interview and has been described in detail elsewhere [16,22]. The FFQ was used to ascertain the frequency of consumption of the four food groups, as recommended by the Ministry of Health’s FNGPB [23] (summarised in Table S1). A number of steps were taken to improve the validity of the FFQ and aid comparison with national data. Questions were formatted to be consistent with 2008/2009 New Zealand Adult National Nutrition Survey [24]. Visual aids were used to assist the reporting of quantities consumed. Description of portion sizes has been demonstrated to increase agreement between FFQ and reference dietary measures [25]. The FFQ was refined during piloting of its content and delivery with a group of pregnant women enrolled around 6 months ahead of the main cohort [26]. The proportion of women meeting recommendations for each food group and the number of food groups met were calculated in women with and without GDM.

Information on diabetes in pregnancy was extracted from the Ministry of Health’s National Minimum Dataset, regional health boards, and laboratories within the recruitment catchment area using participant national health identifiers. Women were categorised as having GDM if coding data included a clinical code for GDM or if laboratory data met the diagnostic criteria for GDM at the time. The New Zealand Society for the Study of Diabetes criteria [27,28] were used by all three regional health boards. These criteria use a 75 g oral glucose tolerance test to diagnose GDM at ≥5.5 mmol/L for fasting glucose or ≥9.0 mmol/L plasma glucose at 2-h post glucose load. One regional health board also considered a 50 g glucose challenge test result of ≥11.1 mmol/L at 60 min post glucose load to be indicative of GDM. Women with pre-existing diabetes or impaired glucose tolerance were excluded from analyses.

**Figure 1:**
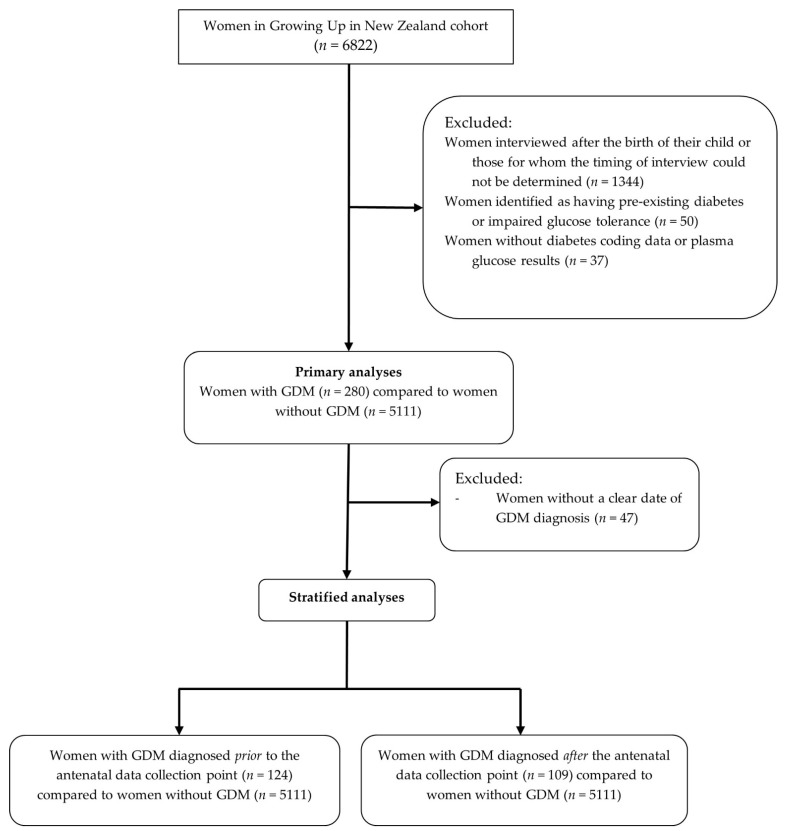
Flowchart showing selection of participants included in primary and stratified analyses of food group recommendation adherence in women with and without GDM from the Growing Up in New Zealand study.

Maternal socio-demographic, health and lifestyle characteristics and adherence to food group recommendations are reported as the frequency (%) for categorical variables and mean (SD) for continuous variables. Cells with *n* <10 are reported as <10 in accordance with GUiNZ policy. Differences in maternal characteristics and adherence to food group recommendations were tested using Chi squared or Fisher’s exact test and unadjusted and adjusted logistic regression. Results are reported as frequency (%), odds ratios (OR) or adjusted odds ratios (aOR) and 95% confidence intervals (CI). Maternal age (<35 and ≥35 years), ethnicity, NZDep06 score (1 to 3, 4 to 7 and 8 to 10), pre-pregnancy BMI (<25, 25 to 29.9 and ≥30 kg/m^2^), pre-pregnancy and first trimester physical activity (engagement in ≥ 150 min of moderate or 60 min vigorous physical activity per week), smoking pattern (continued smoking during pregnancy, stopped smoking during pregnancy, non-smoker), alcohol consumption (continued drinking during pregnancy, stopped drinking during pregnancy, non-drinker) and adherence to food group recommendations were included in adjusted models. The inclusion of variables in adjusted models was based on their association with GDM in univariate or multivariate models or those frequently associated with GDM in the literature. The inclusion and exclusion of participants in this study are shown in Figure 1. Primary analyses were conducted for all women with and without a diagnosis of GDM and stratified analyses were conducted according to the timing of GDM diagnosis in relation to completion of the GUiNZ antenatal data collection interview. Analyses were conducted using SPSS version 21 (IBM Corp., Armonk, NY, USA). A two-sided *p*-value of <0.05 was considered statistically significant.

## 3. Results

Characteristics of women in the GUiNZ cohort have been reported previously [17]. Socio-demographic characteristics of the 5391 women in primary analyses are shown in Table 1. GDM was identified in 280 (5.2%) of women. For those who had a positive diagnosis according to laboratory data (and therefore an ascertainable date of diagnosis), GDM was diagnosed at a mean of 29.4 (6.0) weeks’ gestation. Almost half (44.3%) of women with GDM were diagnosed before the GUiNZ antenatal data collection point and 38.9% of women with GDM were diagnosed after the GUiNZ antenatal data collection point. There were significant differences in a number of maternal characteristics between women with and women without GDM including maternal age, ethnicity, socioeconomic deprivation, pre-pregnancy BMI, gestational weight gain, physical activity, smoking and alcohol consumption (Table 1). Overall, 2.8% of women in the cohort, but 3.2% in women with GDM, adhered to all four food group recommendations (Table 2). Around a quarter of women did not meet the number of servings recommended in any food group.

The number of food groups adhered to was not significantly different between women with and without GDM in primary and stratified analyses (Table 2). The greatest adherence in both women with and without GDM was seen in the proportion of women meeting recommendations for fruit intake (Table 2). Significantly fewer women with GDM met recommendations for milk and milk products and significantly more women with GDM met recommendations for lean meat, poultry, seafood, eggs, nuts and seeds and legumes. When analyses were stratified according to the timing of diagnosis, significantly fewer women with GDM diagnosed prior to the antenatal data collection point (and administration of the FFQ) met the recommended number of servings for fruit, milk and milk products compared to women without GDM and more met the recommendations for lean meat, poultry, seafood, eggs, nuts and seeds and legumes (Table 2). There were no significant differences in the number of women meeting the recommendations for any food group when analyses were restricted to only women with GDM diagnosed after the antenatal data collection point and women without GDM.

Meeting the recommended number of servings for milk and milk products was associated with a significantly reduced odds of developing GDM in the unadjusted model (Table 3). In contrast, meeting the recommended number of servings for lean meat, poultry, seafood, eggs, nuts and seeds and legumes was associated with a significantly increased odds of having GDM. In unadjusted stratified analyses of women with a diagnosis before the antenatal data collection point, meeting the recommendations for the number of servings of fruit also was associated with a reduced risk of developing GDM, but no significant associations were present in unadjusted analyses including only women with a diagnosis of GDM made after the antenatal data collection point (and therefore unlikely to be aware of their forthcoming diagnosis of GDM at the time of data collection) (data not shown). After adjustment, these associations diminished and were no longer significant in both primary and stratified analyses. Meeting any number of food group recommendations did not significantly influence odds of having GDM in unadjusted or adjusted analyses in both primary and stratified analyses (Table 3).

## 4. Discussion

Overall adherence to the Ministry of Health’s FNGPB [23] was poor in women with and without GDM. Less than 4% of women met recommendations for all food groups. Significantly fewer women with GDM reported consuming the recommended number of servings for milk and milk products and more consumed the recommended number of servings for lean meat, poultry, seafood, eggs, nuts and seeds and legumes compared to women without GDM; however, in stratified analysis, this was only the case in women whose diagnosis was made prior to the antenatal data collection point (and therefore presumably aware of their diagnosis at the time). Women whose diagnosis of GDM was made after the antenatal data collection point and who, therefore, were presumably unaware of their impending diagnosis, met recommendations for food groups in similar proportions to women without GDM. This suggests women with a diagnosis of GDM made prior to the GUiNZ antenatal data collection had made adaptations to their diet prior to completing the FFQ, perhaps as a result of their diagnosis or on receiving dietary advice for the management of GDM. In New Zealand, women with GDM are referred to a specialist Diabetes in Pregnancy Team which typically includes an obstetrician, diabetes physician, diabetes midwife and dietitian [29]. Many have already received nutrition advice prior to dietetic input at the Diabetes in Pregnancy Clinic [30]. There were no significant associations between meeting any food group or any number of food groups in adjusted analyses in both primary or stratified analyses. While many studies have found associations between diet and GDM, [31] our data show no significant relationship between meeting recommendations for the number of servings for different food groups and risk of GDM.

In a study using the Australian Recommended Food Score (ARFS), a measure of diet quality according to Australian dietary guidelines, Gresham et al. (2016) found women with higher ARFS had a lower risk of developing gestational hypertension but not of developing GDM. Women with GDM did, however, have a higher mean score for the vegetable component of the ARFS compared to women without GDM [32]. Given the FFQ used by Gresham et al. could have been completed by women up until the time of the birth of their baby, this finding may be due to a treatment affect as seen in our results. Gicevic et al. (2018) explored whether different measures of diet diversity and diet quality could predict the risk of developing GDM in a group of 21,312 women participating in the Nurses’ Health Study II [33]. There were no associations between scores on two diet diversity measures and the risk of GDM; however, higher scores on both the Alternate Health Eating Index-2010 and the Prime Diet Quality Score were associated with a significantly reduced risk of GDM [33]. Similarly, a study of 1489 women participating in the Tongji Maternal and Child Health Cohort study reported a higher score on the newly developed Chinese Dietary Guidelines Compliance Index for Pregnant Women was associated with a reduced risk of GDM [15]. These scores of diet quality provide a more comprehensive assessment of diet quality, as scoring is based on both positive and negative dietary components with scores added for “healthy” foods and subtracted for “unhealthy” foods or nutrients. The scores of diet diversity are a cruder measure of dietary intake as only positive scores are awarded based on consumption of the different food groups [33] and scores do not take into account intake of “unhealthy” food items, similar to measures of adherence to food group recommendations used in our study. In contrast to the findings reported here, analyses of dietary patterns in the same cohort of women in GUiNZ found significant differences in mean dietary pattern scores between women with and without GDM [34]. Given that dietary pattern scores consider the eating pattern as a whole, whilst measures of adherence to food and nutrition recommendations consider only those foods recommended by the guidelines, it may be that such measures of diet quality are not sensitive enough to predict the risk of GDM.

The findings reported here highlight poor adherence to food and nutrition recommendations by pregnant women in New Zealand, despite the majority of women reporting receiving dietary information leading to dietary changes during pregnancy [34]. Our findings are consistent with a recent study exploring adherence to food recommendations in 313 women with GDM in New Zealand which found no woman to meet all food recommendations [35]. Although pregnant women are often thought to be amenable to improving health behaviours during pregnancy [36,37], numerous studies have shown that the quality of women’s diets during pregnancy is poor [38,39]. A survey of 400 pregnant women in Australia found that 65% of women were not familiar with the Australian Guide to Healthy Eating for pregnancy and reported limited differences in women’s nutrition knowledge according to whether women had accessed a dietitian/nutritionist [40]. There are no reports of women’s knowledge of dietary guidelines in New Zealand; however, there are reports indicating that women in New Zealand do make dietary changes during pregnancy [34,41,42]. A survey of 458 women in New Zealand found that, although some women reported using the Ministry of Health’s FNGPB, midwives were the most influential source of dietary information during pregnancy, with over 75% of women reporting receiving dietary advice from their lead maternity carer, consistent with findings previously reported in the same cohort of women included in this study [34]. Clearly, these healthcare professionals play an important role in informing women about the dietary guidelines; however, nutrition is only one of many topics midwives are expected to cover when caring for pregnant women. Internationally, surveys of midwives have expressed a lack of knowledge and confidence in providing nutrition education [43,44]. In New Zealand, midwives report limited formal nutrition education [45] and desire more support in the delivery of nutrition advice [46]. Although New Zealand midwives use the New Zealand Ministry of Health’s FNGPB to inform their nutrition knowledge [45] current strategies for nutrition education in pregnant women clearly are not sufficiently effective in influencing behaviour change. Whilst food and nutrition guidelines are valuable in providing evidence-based advice, further work is needed in their implementation and evaluation in promoting behaviour change. Dietitians are trained to evaluate scientific evidence about food and nutrition and translate it into practical strategies to help people improve their health and lifestyle through nutrition [47]. Early management of GDM and regular contact with a dietitian has been associated with measurable changes in diet [48]. In New Zealand, not all women with GDM are seen by dietitians, and many are seen only once [30]. Whether greater input from a dietitian leads to improved diet and pregnancy outcomes in women with GDM warrants further investigation.

This is the first study describing differences in adherence to dietary guidelines between women with and without GDM in New Zealand. Strengths include the large, ethnically diverse sample, the ascertainment of GDM diagnosis through a number of sources and stratification of analyses according to timing of GDM diagnosis in relation to completion of the FFQ. A limitation is that the FFQ administrated examined dietary intake over just 4 weeks and has not been validated in this population. The broad groupings of foods included in each food group recommendations may limit the usefulness of using adherence to these guidelines to determine differences in risk of GDM given that different foods included in the same food group recommendation may have opposing associations with GDM risk. For example, nuts and seeds and red meat are included in the same recommendation but have been found to be associated with a reduced and increased risk of GDM respectively [49]. A further limitation is that the data collected for the analyses conducted in this study are over 10 years old. Analyses were therefore based on the older Ministry of Health’s FNGPB [23], rather than the Eating and Activity Guidelines for New Zealand adults updated in 2020 to include statements relating to pregnant and breastfeeding women [50]. The Eating and Activity guidelines have different portion size recommendations and recommend a different number of servings for each of the four food groups compared to previous guidelines. Administration of FFQs on a large-scale is both timely and costly. At the time of analyses, the Growing Up in New Zealand study provided the most recent large-scale dataset with both FFQ and GDM diagnosis data available; whether there have been changes to women’s diets and whether there are differences in adherence to these newly published recommendations warrants further investigation.

## 5. Conclusions

In this large, prospective, cohort of pregnant women, adherence to the Ministry of Health’s FNGPB was not significantly associated with the odds of having GDM, most likely due to a lack of sensitivity in using the guidelines as a tool to tease out dietary factors associated with GDM. There were no differences between women with and without GDM in the proportions who met food group recommendations once potential confounding factors were adjusted for. Nevertheless, these findings highlight that pregnant women, even when they have GDM—a condition modifiable by diet—have poor adherence to dietary recommendations. This could lead to poor health for both mother and baby. Differences in adherence to food groups in women with GDM according to timing of diagnosis suggest women make changes to their diet either as a result of their diagnosis or on receiving advice for their diagnosis. Therefore, more support than what is currently provided, for example, more support from a dietitian, may lead to greater adherence to food and nutrition recommendations. Further research on how this can best be achieved and whether the update to the dietary guidelines for pregnant women yields different results is needed.

## Figures and Tables

**Table 1:** Characteristics of women in the Growing Up in New Zealand cohort according to gestational mellitus (GDM) diagnosis ^†^.

*n* (%)	Women without GDM	Women with GDM	*p*-Value
	5111 (94.8)	280 (5.2)	
Age group (years)			<0.005
<20	259 (5.1)	<10 (<10)	
20–24	747 (14.6)	28 (10.0)	
25–29	1267 (24.8)	51 (18.2)	
30–34	1662 (31.7)	95 (33.9)	
35–39	1039 (20.3)	81 (28.9)	
40 and over	177 (3.5)	20 (7.1)	
Self-prioritised ethnicity			<0.005
European	2915 (57.1)	103 (36.8)	
Māori	690 (13.5)	22 (7.9)	
Pacific	631 (12.4)	55 (19.6)	
Asian	681 (13.3)	84 (30.0)	
Other	186 (3.6)	16 (5.7)	
Parity			0.619
First child	2168 (42.4)	123 (43.9)	
Socioeconomic deprivation			0.021
1 to 2 (least deprived)	865 (16.9)	30 (10.7)	
3 to 4	978 (19.1)	45 (16.1)	
5 to 6	909 (17.8)	61 (21.8)	
7 to 8	1052 (20.6)	63 (22.5)	
9 to 10 (most deprived)	1305 (25.5)	81 (28.9)	
Pre-pregnancy BMI (Kg/m^2^)			<0.005
<18.5	192 (4.2)	<10 (<10)	
18.5 to 24.9	2559 (56.2)	103 (41.4)	
25 to 29.9	1034 (22.7)	60 (24.1)	
≥30	772 (16.9)	79 (31.7)	
Gestational weight gain			
Gained ≥5 kg	4460 (88.9)	229 (83.0)	
Gained <5 kg	377 (7.5)	32 (11.6)	
No change	43 (0.9)	<10 (<10)	
Lost <5 kg	74 (1.5)	10 (3.6)	
Lost ≥5 kg	62 (1.2)	<10 (<10)	
Physical activity ^‡^			
Physically active pre-pregnancy	2583 (50.5)	119 (42.5)	0.009
Physically active during first trimester	1464 (28.6)	62 (22.1)	0.019
Physically active during second & third trimester	1152 (22.5)	59 (21.1)	0.567
Smoking patterns			0.012
Continued smoking	509 (10.0)	13 (4.6)	
Stopped smoking	494 (9.7)	31 (11.1)	
Non-smoker	4094 (80.3)	236 (84.3)	
Alcohol consumption			<0.005
Any drinking during pregnancy	1539 (30.1)	42 (15.0)	
Stopped drinking	2279 (44.6)	100 (35.7)	
Non-drinker	1288 (25.2)	138 (49.3)	

^†^ Includes only women interviewed before the birth of their child and excludes women with other forms of diabetes or for whom diabetes status could not be determined; data are presented as the number of participants (%), missing values have not been included in the column %; ^‡^ engaged in moderate or vigorous physical activity for at least 150 or 60 min per week, respectively; BMI, Body Mass Index.

**Table 2:** Adherence to Ministry of Health food group recommendations.

Adherence to Food Group Recommendations	Primary Analyses		Stratified Analyses	
	Women without GDM *n* = 5109	Women with GDM *n* = 280	GDM Diagnosed Prior to FFQ *n* = 124	GDM Diagnosed after FFQ *n* = 109
Four food groups	144 (2.8)	<10 (<10)	<10 (<10)	<10 (<10)
Three food groups	517 (10.1)	31 (11.1)	<10 (<10)	13 (11.9)
Two food groups	1295 (25.3)	68 (24.3)	32 (25.8)	27 (24.8)
One food group	1954 (38.2)	97 (34.6)	49 (39.5)	33 (30.3)
No food groups	1199 (23.5)	75 (26.8)	32 (25.8)	31 (28.4)
Fruit	4245 (83.1)	228 (81.4)	91 (73.4) *	95 (87.2)
Vegetables	1382 (27.0)	87 (31.1)	37 (29.8)	35 (32.1)
Breads and cereals	1350 (26.4)	66 (23.6)	25 (20.2)	25 (22.9)
Milk and milk products	2986 (58.4)	140 (50.0) *	57 (46.0) *	60 (55.0)
Lean meat, poultry, seafood, eggs, nuts and seeds and legumes	1073 (21.0)	81 (28.9) **	36 (29.0) *	30 (27.5)

Data presented as number of participants (%); * *p* < 0.05; ** *p* < 0.005 from Chi squared analyses.

**Table 3:** Unadjusted and adjusted odds of having GDM according to adherence Ministry of Health food group recommendations.

	Primary Analyses		Stratified Analyses	
Adherence to Food Group Recommendations	OR (CI) *n* = 5391	aOR (CI) *n* = 4784	GDM Diagnosed Prior to FFQ and Women without GDM aOR (CI) *n* = 4647	GDM Diagnosed After FFQ and Women without GDM aOR (CI) *n* = 4629
Four vs. at least three food groups	1.15 (0.58, 2.27)	0.88 (0.41, 1.89)	0.69 (0.44, 1.10)	1.46 (0.78, 2.73)
At least three vs. at least two food groups	1.12 (0.80, 1.58)	1.12 (0.77, 1.62)	1.24 (0.81, 1.90)	1.32 (0.84, 2.06)
At least two vs. at least one food groups	1.01 (0.79, 1.30)	1.07 (0.81, 1.41)	0.60 (0.35, 1.00)	0.80 (0.48, 1.34)
At least one vs. no food groups	0.84 (0.64, 1.10)	0.95 (0.70, 1.28)	0.89 (0.60, 1.33)	0.95 (0.62, 1.46)
Four vs. no food groups	1.00 (0.49, 2.04)	0.77 (0.34, 1.77)	1.30 (0.83, 2.04)	1.06 (0.65, 1.75)
Fruit	0.89 (0.66, 1.22)	1.00 (0.70, 1.43)	0.69 (0.44, 1.10)	1.46 (0.78, 2.73)
Vegetables	1.22 (0.94, 1.58)	1.24 (0.93, 1.66)	1.24 (0.81, 1.90)	1.32 (0.84, 2.06)
Breads and cereals	0.86 (0.65, 1.14)	0.82 (0.59, 1.14)	0.60 (0.35, 1.00)	0.80 (0.48, 1.34)
Milk and milk products	0.71 (0.56, 0.91) *	0.93 (0.71, 1.22)	0.89 (0.60, 1.33)	0.95 (0.62, 1.46)
Lean meat, poultry, seafood, eggs, nuts and seeds and legumes	1.53 (1.17, 2.00) **	1.21 (0.88, 1.65)	1.30 (0.83, 2.04)	1.06 (0.65, 1.75)

aOR (95% CI) from adjusted logistic regression (maternal age group, ethnicity, socioeconomic deprivation, pre-pregnancy BMI, pre-pregnancy and 1st trimester physical activity, smoking, alcohol consumption and adherence to food group servings); * *p* < 0.05; ** *p* < 0.005.

## Data Availability

Restrictions apply to the availability of these data. Data was obtained from Growing Up in New Zealand and are available from the authors with the permission of Growing Up in New Zealand.

## Supplementary Materials

The following supporting information can be downloaded at: https://www.mdpi.com/article/10.3390/nu14102145/s1, Table S1: Number of servings for each food group recommended by the Ministry of Health Food and Nutrition Guidelines for pregnant women.
