# Supplementary material for: Adherence to Dietary Guidelines among Women with and without Gestational Diabetes: Evidence from the Growing up in New Zealand Study
Source: Nutrients. 2022 May 21;14(10):2145. doi: 10.3390/nu14102145 (PMC9144046; doi:10.3390/nu14102145)
Supplement: Supplementary file 1 [file nutrients-14-02145-s001.zip › nutrients-1717580-supplementary.pdf]

Table S1. Number of servings for each food group recommended by the Ministry of Health Food and Nutrition Guidelines for pregnant women

Food group	Number of servings recommended per day	Examples of serving size	Specific foods included or excluded from analyses of adherence to food group recommendations
**Fruit and vegetables**<br>(includes fresh, frozen, canned and dried)	≥ 2 fruit and ≥ 4 vegetables<br>Only one serving of juice or dried fruit counts towards the total number of servings per day	1 medium (135 g) potato or kūmera<br>½ cup cooked, salad or mixed vegetables<br>1 apple, pear, banana (130 g)<br>2 small apricots or plums (100 g)<br>½ cup fresh, stewed, frozen or canned fruit<br>1 cup fruit juice<br>2 tablespoons dried fruit	Hot chips, French fries, wedges or kūmara chips not included†<br>Only 1 serving of juice or 1 serving of dried fruit were counted in the total sum of intake
**Breads and cereals**<br>(includes breakfast cereals, breads, grains, rice and pasta, preferably wholegrain)	≥ 6	1 bread roll (50 g)<br>1 medium slice of bread (26 g)<br>½ cup muesli<br>½ c cooked porridge<br>1 cup cooked rice or pasta	Cakes and biscuits not included
**Milk and milk products**<br>(includes milk, cheese, yoghurt and ice-cream and alternatives. Advised to choose low or reduced fat options.)	≥ 3	1 cup of milk<br>1 pot of yoghurt (150 g)<br>2 slices of cheese (40 g)<br>2 scoops of ice-cream	
**Lean meat, poultry, seafood, eggs, nuts and seeds and legumes**	≥ 2	2 slices cooked meat (100 g)<br>¾ cup mince or casserole (195 g)<br>1 egg<br>2 chicken drumsticks or 1 chicken thigh (110g)<br>¾ cup cooked dried beans, peas or lentils<br>½ cup nuts or seeds	Does not include chicken nuggets, chicken roll, processed meats, battered of fried fish, or food prepared in fast-food outlets

†French fries are long thinly cut slices of potato, hot chips are thickly cut slices of potato and kūmara chips are New Zealand native potato fried in fat.
